# Synthesis of hyaluronic acid hydrogels by crosslinking the mixture of high-molecular-weight hyaluronic acid and low-molecular-weight hyaluronic acid with 1,4-butanediol diglycidyl ether†

**DOI:** 10.1039/c9ra09271d

**Published:** 2020-02-18

**Authors:** Yu Xue, Hongyue Chen, Chao Xu, Dinghua Yu, Huajin Xu, Yi Hu

**Affiliations:** School of Pharmaceutical Sciences, State Key Laboratory of Materials-Oriented Chemical Engineering, Nanjing Tech University Nanjing 210009 China huyi@njtech.edu.cn

## Abstract

High molecular weight hyaluronic acid (HMW-HA) and low molecular weight hyaluronic acid (LMW-HA) were mixed at different ratios and cross-linked with 1,4-butanediol diglycidyl ether (BDDE) to prepare five hyaluronic acid hydrogels A–E. Enzymolysis stability, swelling rate, crosslinking degree, rheological characteristics, BDDE residual rate, surface microstructure, and cytotoxicity of different hydrogels were investigated. The results showed that hydrogel B obtained by 10% HA (w/v, HMW-HA and LMW-HA having a mass ratio of 4 : 1) crosslinking with 1% BDDE (v/v) had stronger *in vitro* anti-degradation ability, better mechanical properties and lower cytotoxicity than those prepared by mixing in different proportions. Hydrogel B has potential applications in regenerative medicine and tissue engineering.

## Abstract 2

High molecular weight hyaluronic acid (HMW-HA) and low molecular weight hyaluronic acid (LMW-HA) were mixed at different ratios and cross-linked with 1,4-butanediol diglycidyl ether (BDDE) to prepare five hyaluronic acid hydrogels A–E.

## Graphical abstract

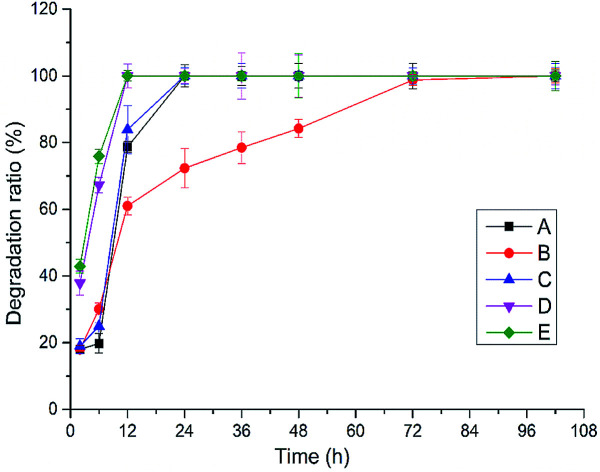

## 1. Introduction

Hyaluronic acid (HA) is a natural glycosaminoglycan and an important component of extracellular matrix, which is biocompatible and biodegradable.^1,2^ The structure and biological characteristics of HA make it have special activity in the process of cell signaling, wound repair and somatic morphogenesis.^3^ Its degradation products have been described as angiogenic and beneficial for the formation of osteoclasts.^4,5^ It also acts as endogenous risk signals in cancer.^6^ HA is widely used in biomedicine, and is a new kind of biological material with practical value. However, native HA networks are relatively weak in mechanical properties, they dissolve rapidly in water and are susceptible to hyaluronidase in the physiological environment.^7^ Physiologically, HA which accounts for one-third of total HA content is degraded and recombined daily in the body.^8^ At the same time, the native HA network will rapidly leave the injection site after injection, and the half-life after injection in the skin and joints is less than 24 h.^9^ All these factors seriously limit the clinical application of HA. Therefore, it is of great significance to modify HA to obtain HA hydrogels with stronger stability and better mechanical properties. HA can be modified by crosslinking,^10^ grafting,^11^ linking with hydrophobic substances,^12^ by forming polyionic complexes with polysaccharides, proteins or surfactants with opposite charges,^13^ or by permeating networks to produce self-assembled aggregates, nanoparticles and gels.^14^ Selin *et al.* proved that the composite material composed of hyaluronic acid and natural polymer could be used as the reinforcement material of tissue engineering scaffolds, it was shown that such composite materials exhibited high thermal and mechanical stabilities.^15^ Angeloni *et al.* prepared a porous chitosan/HA composite and achieved a bone biomimetic scaffold by promoting bone tissue specific protein deposition from differentiated mesenchymal stem cells.^16^ Naumenko *et al.* proposed chitosan scaffolds that are typically doped with other supporting compounds which allow for mechanical strengthening, thus yielding composite biologically resistant scaffolds, to overcome the disadvantages of pure chitosan scaffolds such as mechanical fragility and low biological resistance.^17^

1,4-Butanediol diglycidyl ether (BDDE) can react with HA under strong base conditions to form stable covalent bonds and is a widely used crosslinking agent in the preparation of HA hydrogels. Under alkaline conditions, the epoxy groups of BDDE react preferentially with the hydroxyl group on the primary alcohol in HA.^18^ Stellavato *et al.*^19^ found that when HMW-HA and LMW-HA were physically mixed in a 1 : 1 ratio and heat treated to form HA mixed complexes, their viscosity and *in vitro* resistance to enzymatic hydrolysis were improved. Herein, we hope that a new hydrogel which has strong *in vitro* anti-enzymatic hydrolysis, good mechanical properties and low cytotoxicity could be obtained by crosslinking HMW-HA and LMW-HA in different proportions with BDDE. Swelling rate, enzymatic hydrolysis stability, crosslinking rate, rheological characteristics, BDDE residual rate, surface microstructure, and cytotoxicity were investigated.

## 2. Results and discussion

### 2.1 Enzyme hydrolysis stability

**Fig. 1:**
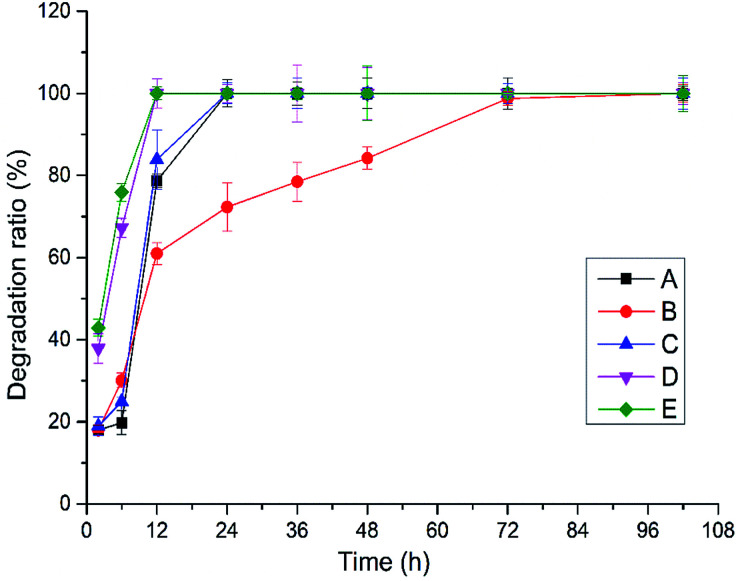
Enzymatic hydrolysis of crosslinked hydrogels A–E prepared at different mixing ratio *in vitro*.

Crosslinked HA hydrogels can be degraded by hyaluronidase from sheep testis, enzymatic hydrolysis condition of hydrogels A–E obtained by BDDE crosslinking with the mixture of HMW-HA and LMW-HA in different ratio was shown in Fig. 1. Hydrogels D and E were completely degraded after 12 h, hydrogels A and C were completely degraded after 24 h, and hydrogels B were completely degraded after 72 h. In addition to hydrogel B, the degradation rate of HA hydrogels increased with the decrease of HWM-HA content, and the degradation rate of HA hydrogel obtained by crosslinking with LMW-HA alone was the fastest. It shows that the mixture ratio of HMW-HA and LMW-HA has a significant effect on the anti-enzymatic hydrolysis performance of the hydrogels. In 10% (w/v) system, the hydrogel B obtained when the mass ratio of HMW-HA and LMW-HA is 4 : 1 has the best anti-enzymatic hydrolysis performance. This phenomenon may be attributed to the reason that the intra HMW-HA chains hydrogen bonds and the intra HMW-HA chains hydrogen bonds have been broken during the preparation process, leading to an entanglement of the H-HA and L-HA chains, which have preferential cooperative bonding between each other.^19^

### 2.2 Swelling ratio

Crosslinked hydrogels are highly water-swollen, they can swell in water under biological conditions, but do not dissolve.^20^ Several characteristics have been exhibited by this high swelling performance, like the low surface tension of surrounding biological liquid and the low mechanical stimulation of surrounding tissue, good diffusion capability including nutriment diffusing into the hydrogel and cell waste removed from the hydrogel.^21^ The ability of molecules to diffuse into or release from expanded hydrogels enables hydrogels to be used in a variety of biomedical applications including in skin enhancement, regenerative drugs, and carriers as bioactive substances.^22^ Therefore, basic measurement of the swelling capacity of hydrogels is critical.

**Fig. 2:**
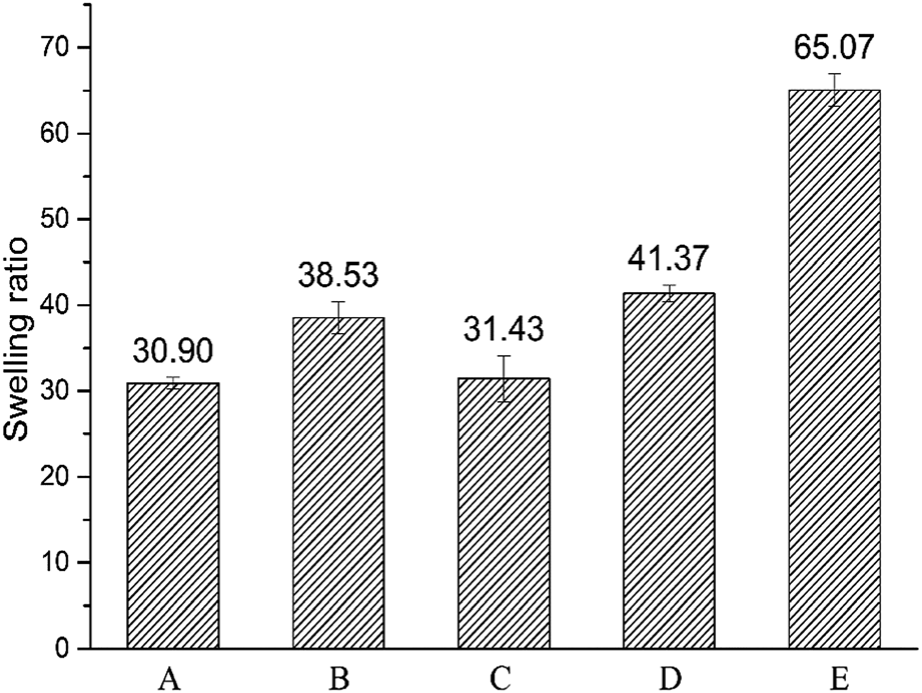
Swelling ratios of crosslinked hydrogels A–E prepared at different mixing ratio in phosphate buffer saline (PBS).

Swelling ratio of crosslinked hydrogels A–E prepared at different mixing ratio in phosphate buffer saline (PBS, pH = 7.2, 0.02 mol l^−1^) was shown in Fig. 2. The different water absorption capacity of hydrogels A–E at equilibrium state indicates that the different mixing ratio of HMW-HA and LMW-HA has certain influence on the expansion performance of hydrogels. The swelling ratio can reflect the degree of modification to a certain extent. The higher the crosslinking degree, the tighter the crosslinking network, and the smaller the swelling rate would be.^23^ As can be seen from Fig. 2, except for hydrogel B, the swelling ratio of HA hydrogels obtained by crosslinking increased with the increase of LMW-HA addition amount.

### 2.3 Determination of crosslinking degree

**Fig. 3:**
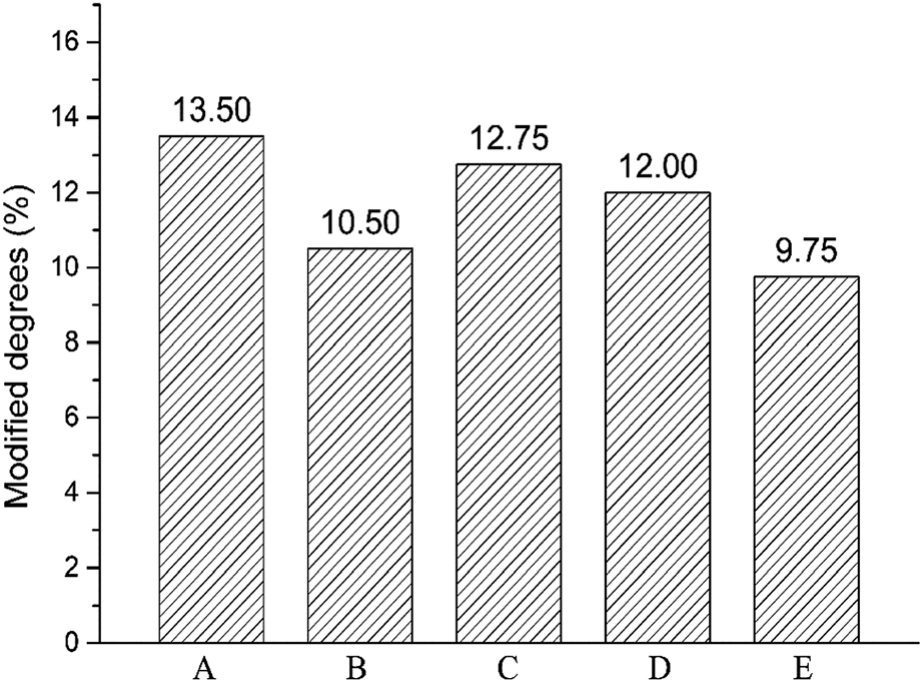
Modified degrees of crosslinked hydrogels A–E prepared at different mixing ratio.

Crosslinking degree of crosslinked hydrogels A–E prepared at different mixing ratio was different and calculated by ^1^H NMR analysis of the hydrolysate. The signal at *δ* = 1.5 ppm belongs to the –(CH_2_) 2 signal peak in the middle of BDDE structure. The signal peak *δ* = 1.8 ppm belongs to the *N*-acetyl group in HA.^24^ The chemical crosslinking degree of crosslinked HA (MoD) was calculated by eqn (1). The results were shown in Fig. 3, and the spectrum were shown in the ESI.†1
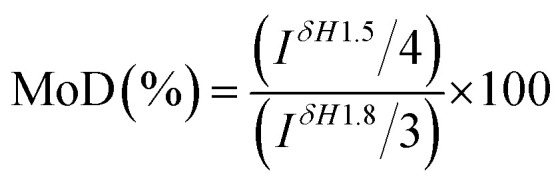


The crosslinking degrees of HA hydrogels were ranked as E, B, D, C, and A in descending order. The result was basically consistent with swelling property in the previous section. In addition to hydrogel B, the modification of hydrogels A–E decreased with the addition of LMW-HA. The crosslinking degree of hydrogel B was significantly lower than that of hydrogel A prepared by HMW-HA alone. This phenomenon may exist for the reason that the addition of LMW-HA will reduce the crosslinking efficiency and the overall mechanical properties of the hydrogel within a certain range.^25^ The high crosslinking degree of hydrogel A may lead to more BDDE residues, which could enhance the potential toxicity to cells.

### 2.4 Rheological experiments

**Fig. 4:**
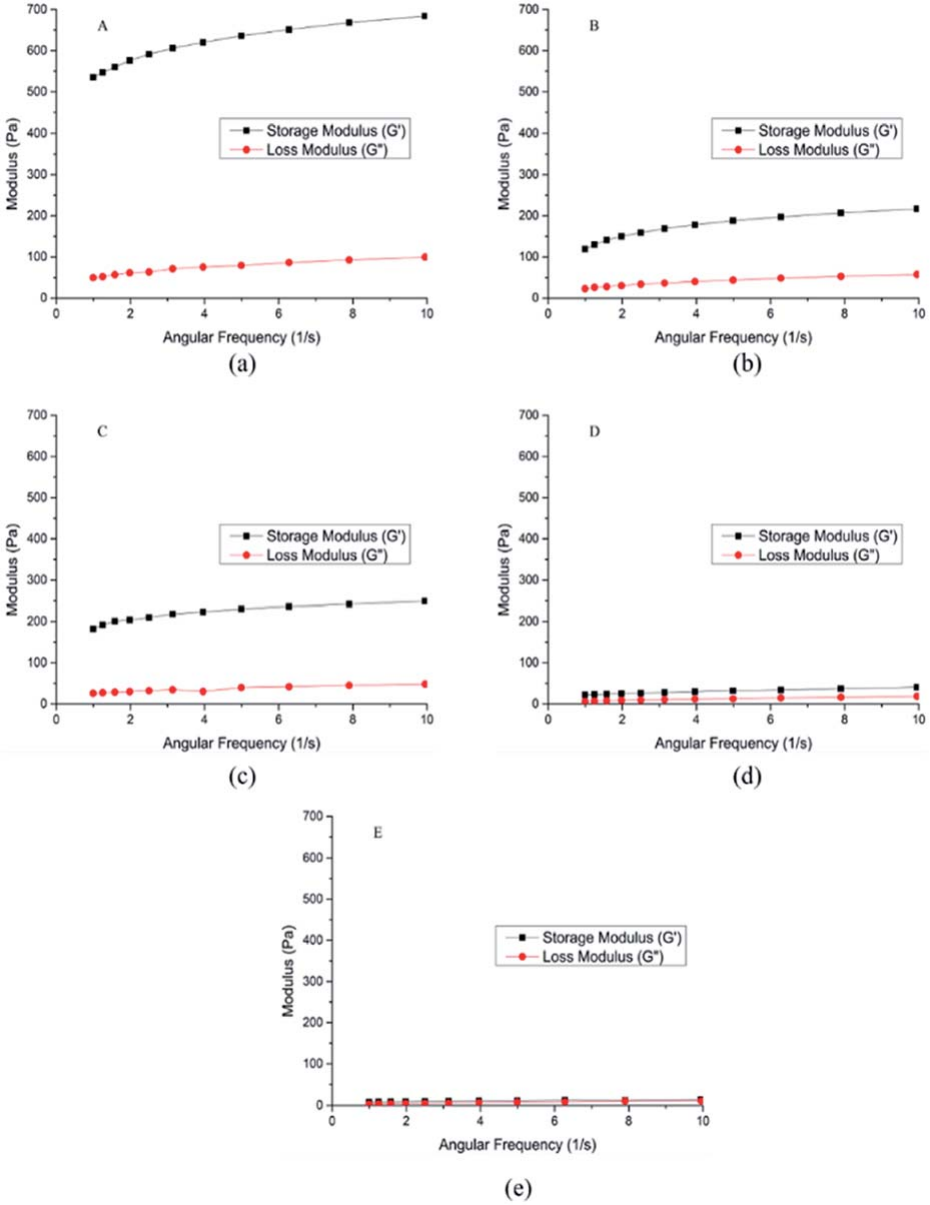
Rheological properties of crosslinked hydrogels A–E prepared at different mixing ratio.

The rheological properties of the hydrogel A–E were shown in Fig. 4, and some parameters were shown in Table 1. Storage moduli (*G*′) of the hydrogel A–E were higher than loss moduli (*G*′′), indicating that the hydrogel A–E had great viscoelasticity.^26^

**Table 1:** Viscoelasticity of crosslinked hydrogels A–E prepared at different mixing ratio

Entry	*G*′ (Pa)		*G*′′ (Pa)	
	1 Hz	10 Hz	1 Hz	10 Hz
A	535.00	684.00	49.70	99.60
B	119.00	217.00	23.00	57.60
C	182.00	249.00	26.00	48.20
D	22.00	40.60	6.62	18.70
E	8.05	14.10	3.23	11.00

The higher the storage modulus is, the greater the stiffness will be, the less deformation the material will be, and the stronger the brittleness will be. The lower the loss modulus is, the larger the deformation is, and the smaller the stiffness is. Therefore, hydrogel A was more brittle than hydrogel B, and hydrogel B was more flexible than hydrogel A, which meant hydrogel A with strong anti-enzymatic hydrolysis ability had better injection performance.^23^ As for D and E, the value of *G*′ was close to the value *G*′′, which represented that D ang E were semisolid with low mechanical strength.

### 2.5 BDDE residues

BDDE has certain biological toxicity and potential carcinogenicity. Therefore, BDDE residues of hydrogels were investigated. The results were shown in Table 2. The BDDE residual amount of the hydrogel B with the best enzymatic hydrolysis performance was lower than that of the hydrogel A.

**Table 2:** The residual amount of BDDE in crosslinked hydrogels A–E prepared at different mixing ratio *in vitro*

Entry	The residual amount of BDDE (μg g^−1^)
A	10.42
B	8.18
C	3.23
D	3.01
E	4.99

### 2.6 Scanning electron microscope (SEM)

**Fig. 5:**
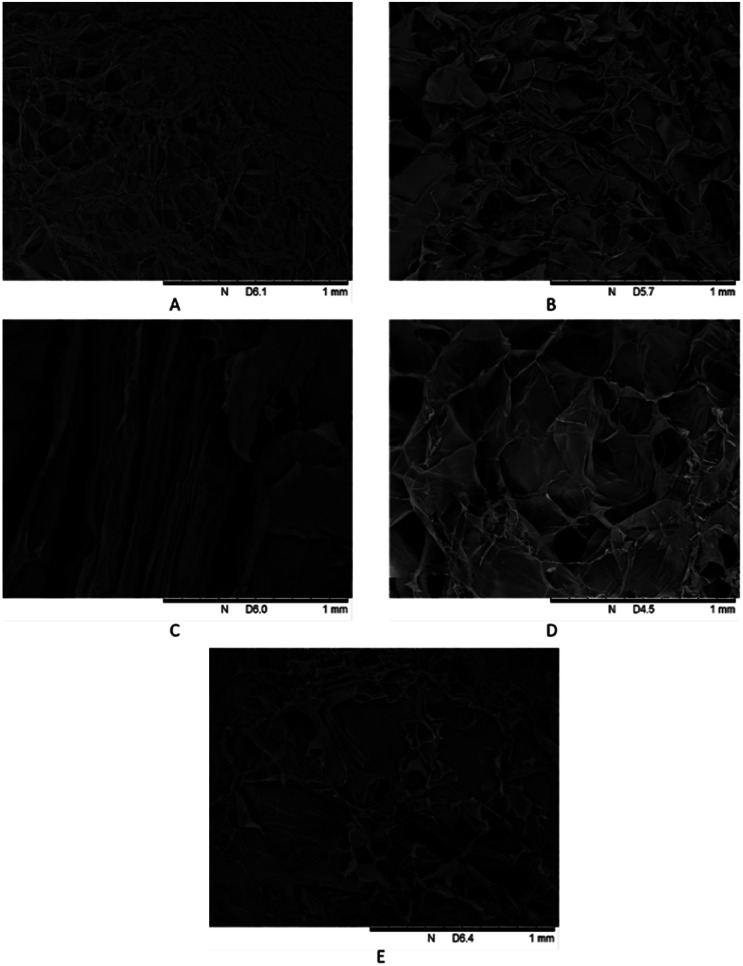
SEM spectra of crosslinked hydrogels A–E prepared at different mixing ratio.

Although SEM cannot determine the presence of ether bonds in crosslinked hydrogels, it provides unique information about porosity and scaffold connectivity. Fig. 5 showed the SEM surface images of hydrogel A–E. SEM images showed the microstructure differences of hydrogels. Hydrogels A, B and D formed dense cross-linking networks with different pore structures. Hydrogels D had the largest pore structure, so it was easy to be hydrolyzed by enzymes. Hydrogel C formed large cracks in the freeze-drying process, possibly because the excessive amount of LMW-HA was added, which resulted in uneven distribution in the crosslinking process. The LMW-HA chain was relatively short and easy to fracture in the freeze-drying process. However, most layers were closely connected with each other, and hydrogel C also had relatively good anti-enzymatic hydrolysis ability. Hydrogel E was closer to the original HA image reported in the literature,^27^ in the process of freeze-drying, the extremely thin layered structure and loose pores were formed, and there was a certain degree of collapse. This may also be one of the reasons for its weak anti-degradation ability.

### 2.7 Cytotoxicity test

From previous sections, a hydrogel with strong *in vitro* anti-degradation ability, low BDDE residue and strong flexibility has been prepared without too much introduction of crosslinking agent (hydrogel B: 10% HA (w/v, HMW-HA and LMW-HA mass ratio 4 : 1), 1% BDDE (v/v)). To evaluate its safety as a potential biomedical material, MTT assay was used to determine its toxicity to mouse L929 fibroblasts. The cytotoxicity of hydrogel A prepared by HMW-HA alone under the same conditions was compared. Cytotoxicity was graded according to the relative cell proliferation rate, as shown in Table 3.

**Table 3:** Relative growth rate and cytotoxicity grade

Relative cell proliferation rate (%)	Cytotoxical grade
≥100	0
75–99	1
50–74	2
25–49	3
1–24	4
≤1	5

As shown in Table 4 and Fig. 8, the cytotoxicity of hydrogel A was grade 1 and grade 2 at most extraction concentrations, and at very low concentration of 0.1 mg ml^−1^, the cytotoxicity was grade 0. The cytotoxicity of hydrogel B extracted at different concentrations was level 0. Compared with the blank control group, hydrogel B extracts at different concentrations promoted the proliferation of L929 cells. In the positive control group, mouse L929 fibroblasts was cultured with phenol diluent, apoptosis was observed and the relative cell proliferation rate dropped to 26.680%, indicating the cytotoxicity was level 3. This indicated that hydrogel B promoted cell viability, which may be due to the small amount of LMW-HA in the leaching solution could bind to specific receptors and stimulate the proliferation of fibroblasts.^28^ It can be clearly seen from Table 5 that the cytotoxicity of hydrogel B was lower than that of hydrogel A.

**Table 4:** The OD values of each test group and the relative growth rate of L929 cells at 24 h

	Entry	OD value (*x̄* ± *S*)	Relative growth rate (%)	Cytotoxicity
Hydrogel A	100 mg ml^−1^	0.199 ± 0.038	75.03	1
	20 mg ml^−1^	0.183 ± 0.019	69.182	2
	5 mg ml^−1^	0.171 ± 0.030	64.340	2
	1 mg ml^−1^	0.199 ± 0.022	75.220	1
	0.5 mg ml^−1^	0.138 ± 0.020	52.138	2
	0.1 mg ml^−1^	0.267 ± 0.027	100.566	0
	Blank control group	0.265 ± 0.028	100.000	0
	Positive control group	0.059 ± 0.016	22.075	4
Hydrogel B	100 mg ml^−1^	0.278 ± 0.044	110.978	0
	20 mg ml^−1^	0.264 ± 0.043	105.522	0
	5 mg ml^−1^	0.386 ± 0.028	153.892	0
	1 mg ml^−1^	0.316 ± 0.023	126.214	0
	0.5 mg ml^−1^	0.463 ± 0.062	184.830	0
	0.1 mg ml^−1^	0.309 ± 0.045	123.420	0
	Blank control group	0.251 ± 0.035	100.000	0
	Positive control group	0.067 ± 0.005	26.680	3

**Table 5:** Composition list of raw material for HA hydrogels

	A	B	C	D	E
HMW-HA (g)	0.5	0.4	0.375	0.333	0
LMW-HA (g)	0	0.1	0.125	0.167	0.5
NaOH (ml)	4.95	4.95	4.95	4.95	4.95
BDDE (μl)	50	50	50	50	50

**Fig. 6:**
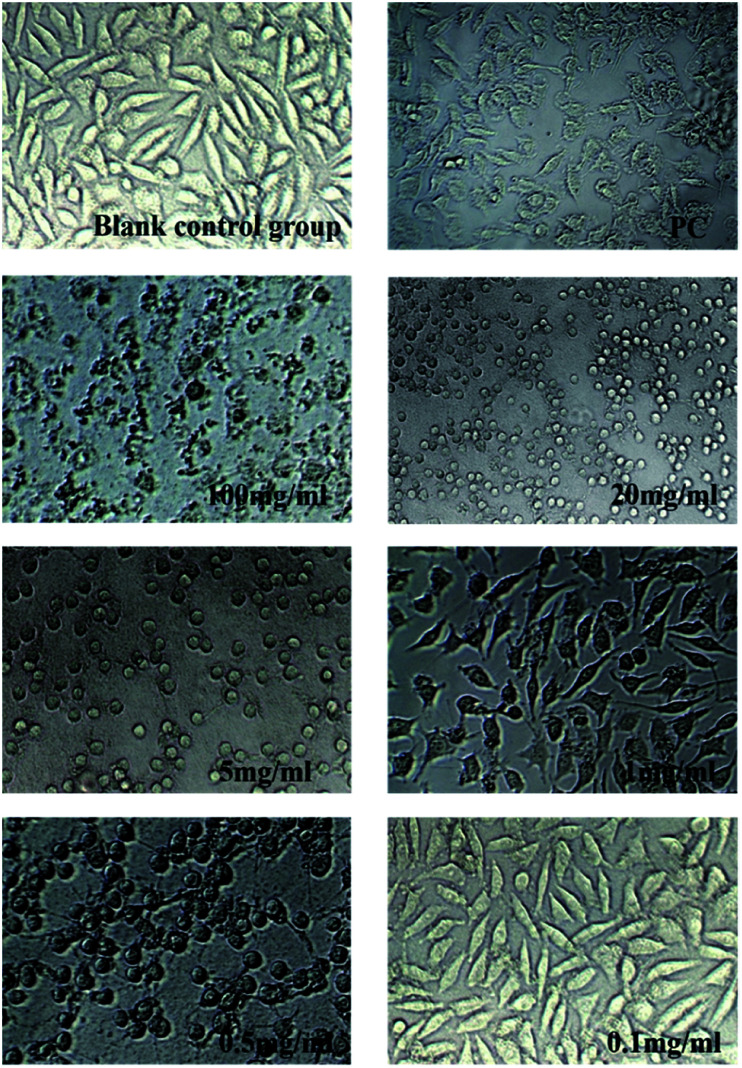
Changes in cell morphology after incubated by Hydrogel A extract for 24 h (×200).

**Fig. 7:**
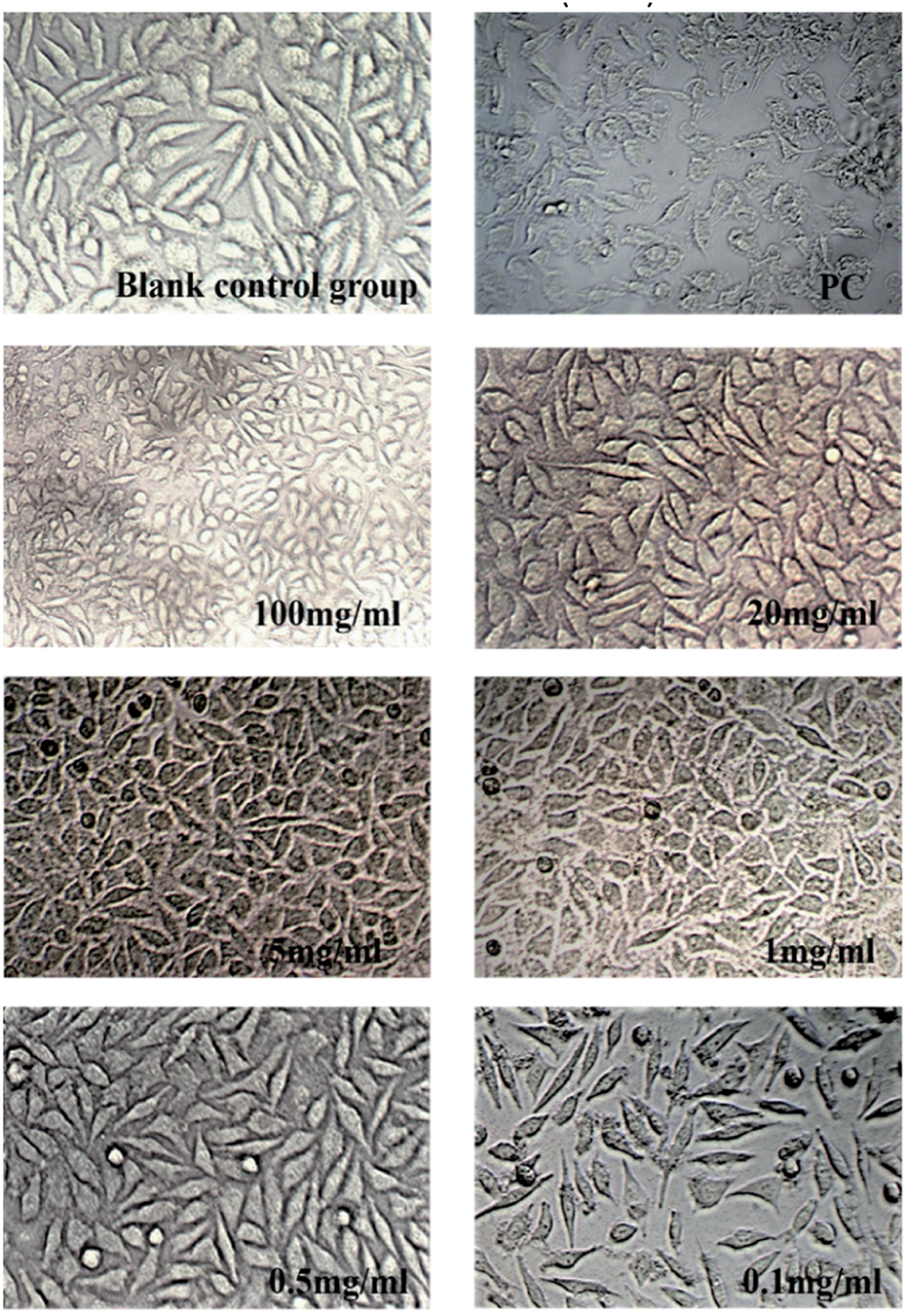
Changes in cell morphology after incubated by Hydrogel B extract for 24 h (×200).

As was shown in Fig. 6 and 7, blank control group L929 cells were flat spindle-shaped and angular, the morphology of cells in the positive control group (PC) was significantly changed, with a small amount of morphology remained, and the majority of cells were atrophic with fuzzy edges. As can be seen from Fig. 6, after incubation in hydrogel A extracts (100 mg ml^−1^, 20 mg ml^−1^, 5 mg ml^−1^, 1 mg ml^−1^ and 0.5 mg ml^−1^) for 24 h, the morphology of L929 cells changed to some extent, resulting in cell atrophy and fuzzy or round edges, indicating that hydrogel A had relatively high biological toxicity. As can be seen from Fig. 7, after 24 h incubation in hydrogel B extract, no significant morphological changes were observed in L929 cells, which were fusiform with clear boundaries and complete morphology, similar to cells in the normal negative control group, preliminarily proving the biosafety of hydrogel B. The results were consistent with the result of cytotoxicity grading.

**Fig. 8:**
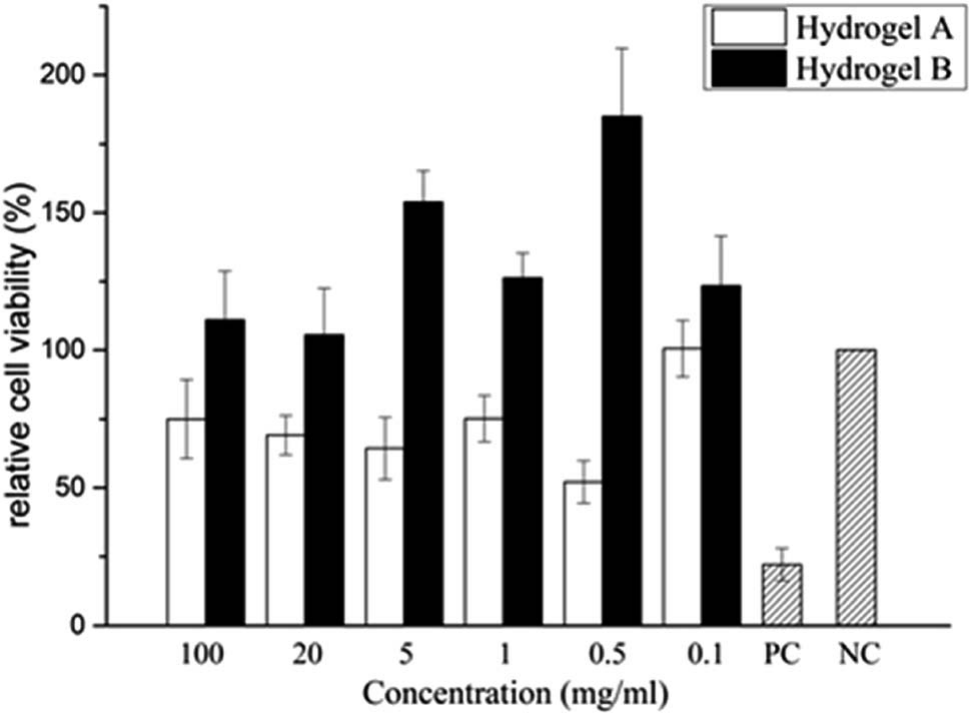
Changes of relative cell viability after incubated by HA hydrogel extract for 24 h.

## 3. Experimental

### 3.1 Materials

The high-molecular-weight HA sample (2000 kDa) and low-molecular-weight sample (200 kDa) were purchased from Bloomage Freda Biopharm Co. Ltd. (Shandong, China); 1,4-butanediol diglycidyl ether (BDDE), (methyl sulfoxide)-d_6_ and d-glucuronic acid were purchased from Meryer (Shanghai) Chemical Technology Co., Ltd. (Shanghai, China); NaOH, KOH, NaH_2_PO_4_·2H_2_O, Na_2_HPO_4_·12H_2_O, sodium tetraborate decahydrate and nicotinamide were purchased from Sinopharm Chemical Reagent Co. Ltd (Shanghai, China); hyaluronidase was purchased from Shanghai yuanye Bio-Technology Co. Ltd. (Shanghai, China); H_2_SO_4_ was purchased from Shanghai Lingfeng Chemical Reagent Co. Ltd (Shanghai, China); carbazole and formic acid were purchased from Shanghai Aladdin Bio-Chem Technology Co. Ltd (Shanghai, China); acetophenone was purchased from Energy Chemical (Shanghai, China). All the chemicals were of analytical grade.

### 3.2 Methods

#### 3.2.1 Preparation of hyaluronic acid hydrogel crosslinked by BDDE

A total of 10% (w/v) HA powders with different ratios of HMW-HA and LMW-HA solution were prepared by dissolving in 0.25 M NaOH with 1% (v/v) BDDE. After reaction at 40 °C for 6 h, the reaction mixture was washed with deionized water 3 times to obtain the hydrogel product. The hydrogel was dialyzed against phosphate buffer (PBS) for 24 h then dialyzed with deionized water for 24 h to fully remove unreacted BDDE, lyophilized in a lyophilizer for 48 h to obtain white spongy samples. The composition list of crosslinked HA hydrogels are shown in Table 5.

#### 3.2.2 Enzyme hydrolysis stability

12 mg lyophilized sample was swelled in PBS for 12 h. The swelling hydrogel was mixed with 10 ml hyaluronidase solution (100 U ml^−1^, dissolved in PBS), and then incubated at 42 °C. 0.5 ml sample was taken at 2 h, 6 h, 12 h, 24 h, 36 h, 48 h, 72 h and 102 h from liquid supernatant. The enzyme in sample was deactivated in a boiling water bath for 10 min. After cooling to room temperature, the sample was filtered through 0.22 μm PTFE membrane, and the concentration of glucuronic acid in the filtrate was determined by Bitter–Muir method.^29^ The glucuronic acid concentration in supernatant was signed as c1 and the final concentration of glucuronic acid in the supernatant was signed as *c*_2_. The degradation ratio was defined as the eqn (2).

2 The degradation ratio (%) = *c*_1_/*c*_2_ × 100

#### 3.2.3 Swelling ratio^30^

Lyophilized sample was immersed in phosphate buffer saline (PBS) and was taken out until equilibrium state. The excess water on the surface was removed by filter paper and sample was re-weighed. Swelling ratio was calculated by eqn (3).

3 Swelling ratio (g g^−1^) = *W*_s_/*W*_d_

*W*_s_ is the weight of sample at equilibrium state and *W*_d_ is the weight of initial lyophilized sample.

#### 3.2.4 Determination of crosslinking degree

Lyophilized sample was hydrolyzed by sulfuric acid solution (0.5 M). The hydrolysate was then lyophilized, dissolved in 6 ml DMSO-d_6_ and transferred into 5 mm NMR tubes. The NMR spectrum was measured on a Bruker Advance 300 MHz instrument (Bruker, Germany) with DMSO-d_6_ as the solvent and TMS as internal standard.

#### 3.2.5 Rheological experiments

Rheological experiments were performed using a Physica MCR302 oscillatory rheometer (Anton Paar, Germany) which equipped with a parallel plate geometry, 25 mm plate diameter, 1.0 mm gap, and a Peltier temperature control. The experiment temperature was 37 °C. The strain value was 0.1% and oscillation frequency sweep tests were carried out over a frequency range from 1 to 10 Hz.

#### 3.2.6 BDDE residues^31^

BDDE residues can be tested by detecting fluorescence intensity of the substance produced by BDDE and nicotinamide, which has strong fluorescent where excitation wavelength and emission wavelength were located at 370 nm and 430 nm. 20 μl 8.0, 4.0, 2.0, 1.0, 0.5, 0.25 μg ml^−1^ BDDE solution and 10 μl 125 mM l^−1^ nicotinamide solution were mixed respectively in tubes and incubated 2 h at 37 °C. 100 μl 15% acetophenone solution (w/w, dissolved in ethanol) and 100 μl 1 mol l^−1^ potassium hydroxide solution were added into tubes, set on ice for 10 min. Then 0.5 ml formic acid was added into tubes and incubated for 5 min at 60 °C. The fluorescence values were determined by Multifunctional microplate reader SpectraMax M5 (Thermo Fisher Science, USA) where excitation wavelength and emission wavelength were located at 370 nm and 430 nm. BDDE concentration and fluorescence were used as the abscissa and the ordinate to make the standard curve. 1 g swelling HA hydrogels were hydrolyzed by 1 ml hyaluronidase solution (100 U ml^−1^). The BDDE content of enzymatic hydrolysate was determined by the method above.

#### 3.2.7 Scanning electron microscope (SEM)

Lyophilized sample was first deposited on aluminum stubs and then coated with gold using an ion sputter and then visualized by scanning electron microscope (SEM) from Hitachi Tabletop Microscope (TM3000, Tokyo, Japan).

#### 3.2.8 Cytotoxicity test

In order to evaluate safety of hydrogels as a potential biomedical material, MTT method has been used to determine the cytotoxicity of hydrogels and the effect on cell morphology.^32^ L929 mouse fibroblasts were purchased from CAS typical culture collections Committee cell library (Shanghai, China). Cells were cultured in minimum essential medium (MEM) with 10% (w/w) FBS and 1% (w/w) penicillin/streptomycin at 37 °C in a humidified atmosphere of 5% CO_2_. Extract liquid was prepared by extracting the hydrogel sample at 37 °C with culture medium for 24 h. L929 mouse fibroblasts (2.5 × 10^4^ cells per cm^2^) were cultured in 96-well microplates for 24 h. Then medium was discarded, cells were treated with 100 mg ml^−1^, 20 mg ml^−1^, 5 mg ml^−1^, 1 mg ml^−1^, 0.5 mg ml^−1^, 0.1 mg ml^−1^ extract liquid for 24 h, respectively. Equal volume of culture medium was used as blank control and equal volume of phenol diluent (1%, v/v) as positive control. Cell morphology was recorded by Leica DMI3000B manual inverted microscope (Leica Microsystems, Germany). Subsequently, cells were stained with MTT at final concentration of 0.5 mg ml^−1^ in PBS (pH 7.4) for 4 h in dark and then the medium was discarded. The formazan crystals presented in cells were dissolved by 100 μl of DMSO. The absorbance was read at 490 nm on a Multiskan MK3 microplate reader (Thermo Fisher Science, USA). The relative growth rate (RGR) was calculated by eqn (4).4
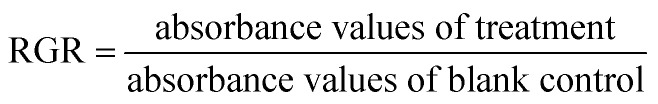


## 4. Conclusions

HA hydrogels were prepared by crosslinking the mixture of HMW-HA and LMW-HA at different ratio with BDDE under alkaline conditions, and compared with that prepared by crosslinking HMW-HA or LMW-HA with BDDE alone. *In vitro* anti-enzymatic hydrolysis, swelling and rheological properties were investigated. The crosslinking degree and residue of crosslinking agent BDDE in hydrogels were determined, and the surface morphology was investigated by SEM. A novel crosslinked HA hydrogel B with strong *in vitro* anti-degradation ability, low BDDE residue and strong mechanic strength and flexibility could be obtained by 10% HA (w/v, HMW-HA and LMW-HA have a mass ratio of 4 : 1) crosslinking with 1% BDDE (v/v). The cytotoxicity of hydrogel B was determined by MTT assay, the effect of the extracts on cell morphology was investigated, and the hydrogel A obtained by HMW-HA crosslinking with BDDE under the same conditions was compared. The results showed that the cytotoxicity level of hydrogel B was 0, which promoted the proliferation of L929 cells to a certain extent, and had no significant effect on the morphology of L929 cells. Hydrogel B has better biological safety than hydrogel A, and can be used as potential biomaterials in biomedical and other fields.

## Conflicts of interest

There are no conflicts to declare.

## Supplementary Material

RA-010-C9RA09271D-s001
