# Supplementary material for: Synthesis of hyaluronic acid hydrogels by crosslinking the mixture of high-molecular-weight hyaluronic acid and low-molecular-weight hyaluronic acid with 1,4-butanediol diglycidyl ether
Source: RSC Adv. 2020 Feb 18;10(12):7206–13. doi: 10.1039/c9ra09271d (PMC9049836; doi:10.1039/c9ra09271d)
Supplement: RA-010-C9RA09271D-s001 [file RA-010-C9RA09271D-s001.pdf]

Electronic Supplementary Material (ESI) for RSC Advances.
This journal is © The Royal Society of Chemistry 2020

Supplementary material

1H NMR spectra of HA hydrogel hydrolyzed product

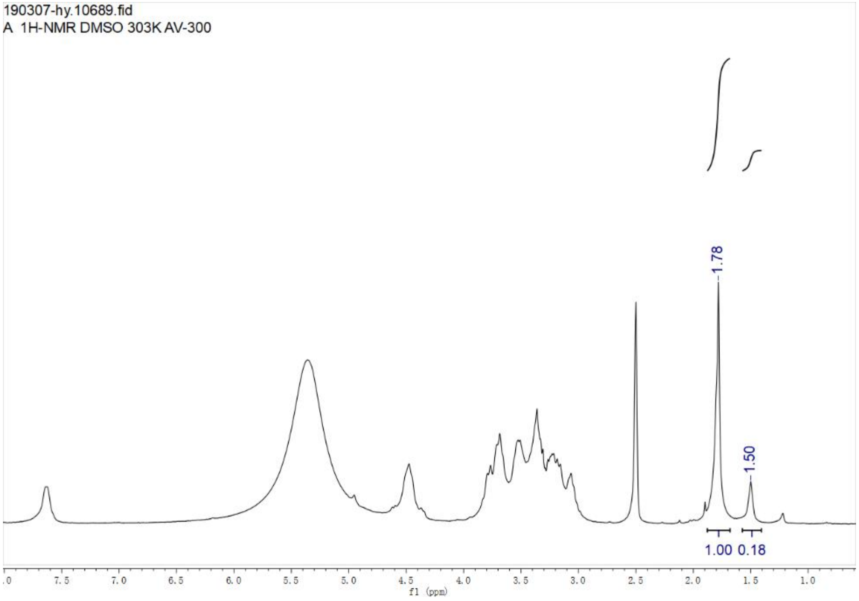

A

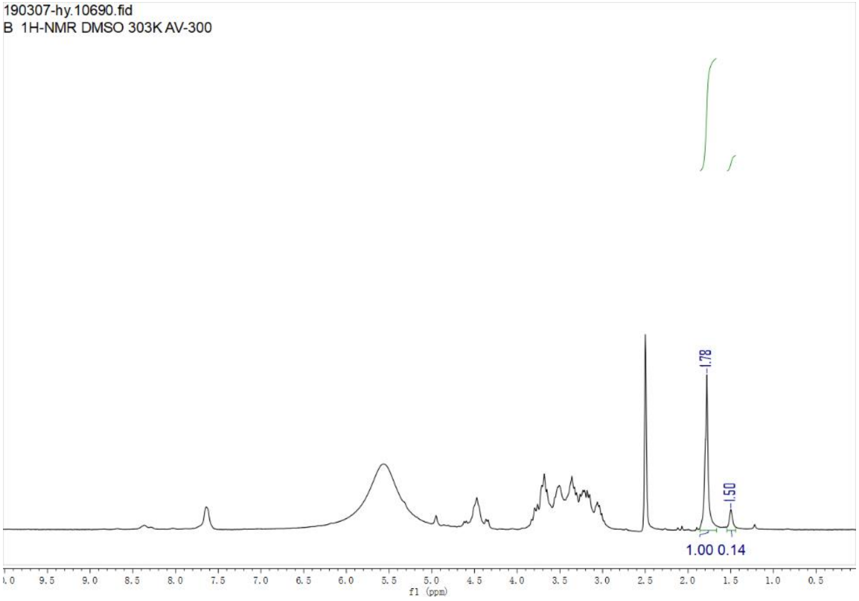

B

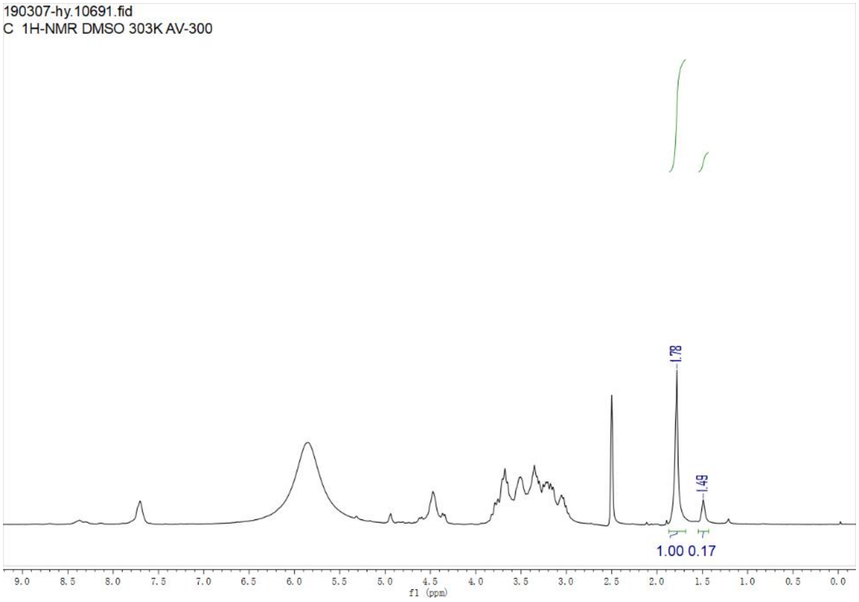
190307-hy.10691.fid
C 1H-NMR DMSO 303K AV-300
1.78
1.49
1.00 0.17
9.0 8.5 8.0 7.5 7.0 6.5 6.0 5.5 5.0 4.5 4.0 3.5 3.0 2.5 2.0 1.5 1.0 0.5 0.0
f1 (ppm)

C

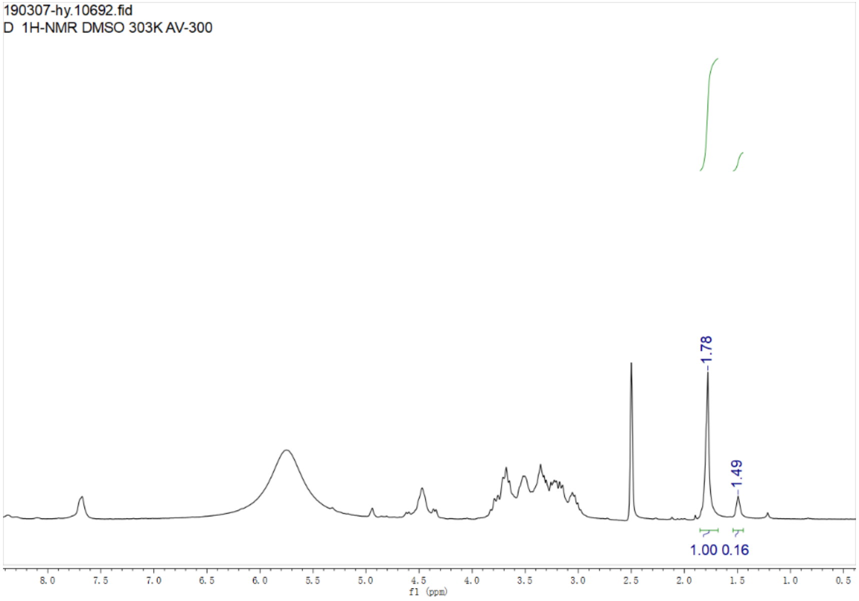
190307-hy.10692.fid
D 1H-NMR DMSO 303K AV-300
1.78
1.49
1.00 0.16
8.0 7.5 7.0 6.5 6.0 5.5 5.0 4.5 4.0 3.5 3.0 2.5 2.0 1.5 1.0 0.5
f1 (ppm)

D

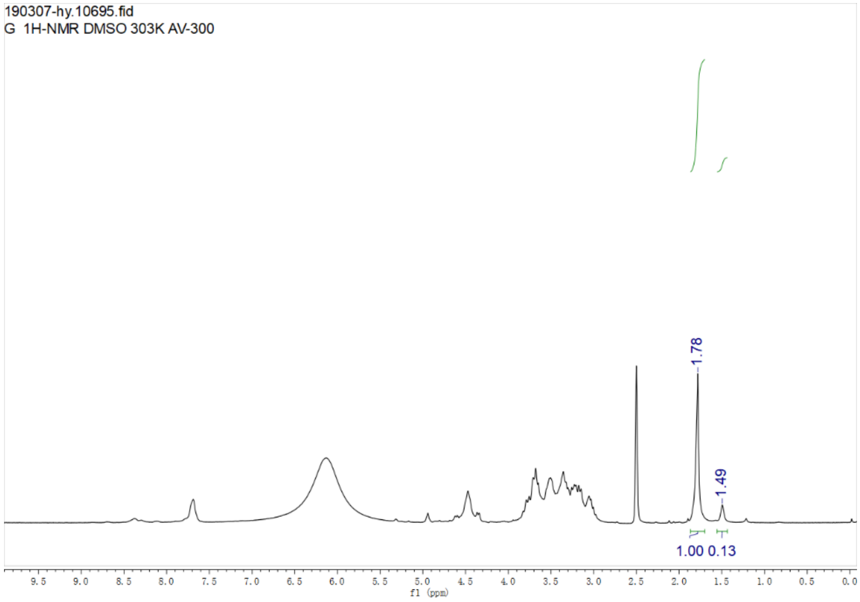
190307-hy.10695.fid
G 1H-NMR DMSO 303K AV-300
1.78
1.49
1.00 0.13
9.5 9.0 8.5 8.0 7.5 7.0 6.5 6.0 5.5 5.0 4.5 4.0 3.5 3.0 2.5 2.0 1.5 1.0 0.5 0.0
f1 (ppm)

E
